# Altered small-world property of a dynamic metabolic network in murine left hippocampus after exposure to acute stress

**DOI:** 10.1038/s41598-022-07586-6

**Published:** 2022-03-10

**Authors:** Min-Hee Lee, Yoon Ho Hwang, Chang-Soo Yun, Bong Soo Han, Dong Youn Kim

**Affiliations:** 1 Institute of Human Genomic Study, College of Medicine, Korea University Ansan Hospital, Ansan, Republic of Korea; 2 Department of Biomedical Engineering, Yonsei University, Wonju, Republic of Korea; 3 Department of Radiation Convergence Engineering, Yonsei University, Wonju, Republic of Korea

**Keywords:** Computational neuroscience, Stress and resilience

## Abstract

The acute stress response is a natural and fundamental reaction that balances the physiological conditions of the brain. To maintain homeostasis in the brain, the response is based on changes over time in hormones and neurotransmitters, which are related to resilience and can adapt successfully to acute stress. This increases the need for dynamic analysis over time, and new approaches to examine the relationship between metabolites have emerged. This study investigates whether the constructed metabolic network is a realistic or a random network and is affected by acute stress. While the metabolic network in the control group met the criteria for small-worldness at all time points, the metabolic network in the stress group did not at some time points, and the small-worldness had resilience after the fifth time point. The backbone metabolic network only met the criteria for small-worldness in the control group. Additionally, creatine had lower local efficiency in the stress group than the control group, and for the backbone metabolic network, creatine and glutamate were lower and higher in the stress group than the control group, respectively. These findings provide evidence of metabolic imbalance that may be a pre-stage of alterations to brain structure due to acute stress.

## Introduction

Acute stress is a physiologically or biologically adaptive response to one extreme stressor that threatens homeostasis^1,2^. It is important to understand the stress response because it is a fundamental natural mechanism for survival that protects the immune system and improves functional performance from external stimuli^3^. The stress response, which involves some, neurotransmitters, and neuropeptides, occurs over time, ranging from tens of minutes to hours^4,5^. The effects of the stress response manifest themselves in two ways: short-term and long-term. Time-dependent variation of neurochemicals, including the stress hormones, necessitates investigation of the metabolic changes over time^2^. If the stress response is maladaptive over time, it causes structural and functional changes in the brain^6,7^.

The brain is a central mediator that regulates the stress response^8^. Since the hippocampus is involved in the regulation of the hypothalamic-pituitary-adrenal (HPA) axis, which regulates stress hormones (i.e., corticotropin-releasing hormone and glucocorticoids) and neurotransmitters by negative feedback, the hippocampus is an important stress-related region and vulnerable to acute stress^9,10^. Because of the difference in the function and balance of stress hormones and the interaction of neurotransmitters, the brain’s ability to regulate and successfully adapt to acute stress, called stress resilience, varies between individuals^4^. Stress resilience is important and is necessary for a variety of situations that can cause acute stress with the following associations: 1) quick activation and successful termination of the stress response^11^ and 2) the ability to restrain the increase of stress hormones and neurotransmitters due to acute stress through a sophisticated negative feedback^11,12^. If not, the brain is vulnerable to acute stress, which induces a reorganization of brain architecture, which may be associated with the onset and exacerbation of several neuropsychiatric disorders^13^. Considering these findings, it can be concluded that studies on time-dependent responses to acute stress are necessary, and they can be analyzed through temporal changes in neurotransmitters as opposed to in previous studies^14,15^ that compared the changes of neurotransmitters with a focus on consequential changes, which can cause bias.

To investigate the changes in neurotransmitters over time, a proton magnetic resonance spectroscopy ($^1$H-MRS) is used to quantify neurotransmitters called brain metabolites in the desired brain regions. Many researchers have employed $^1$H-MRS, which facilitates quantification of the metabolites to investigate the effect of acute stress on the brain at a metabolic level, since the alteration of neurogenesis and neurochemistry can be induced by acute stress alone^9^. Using metabolic variation and changes in the concentration of metabolites over time, the relation between the metabolites, which may be used as a measurement of the similarity of metabolite conditions, profiles, and biological information, can be determined with analyzable values. It is important to analyze the alteration of metabolic relationship because it can provide insights for understanding the dynamics of metabolic response to acute stress^16^. Since the conventional $^1$H-MRS approach cannot determine the relation between metabolites, a novel approach is needed.

A real-world complex system in a multidisciplinary research area can be modeled as a network^17^, such as a social network^18^, an urban road network^19^, a biological network^20^, and a brain network^21^. A network analysis, based on the graph theoretical approach, which represents relations between discrete objects, is a quantitative and computable measure of network organization^22^ that describes the connections of the metabolites as a collection of nodes (i.e., metabolites) and edges (i.e., metabolite-metabolite relationships) between pairs of nodes in the present study. In the network analysis, the small-worldness (SW) proposed by Watts and Strogatz^23^ may be employed to evaluate whether the network is a realistic network relative to a random network^24^ and whether the network balances global integration and local segregation for efficient information processing^25^. This study presumes that network analysis can provide effective and comprehensive information for the process of metabolic response to acute stress.

**Figure 1:**
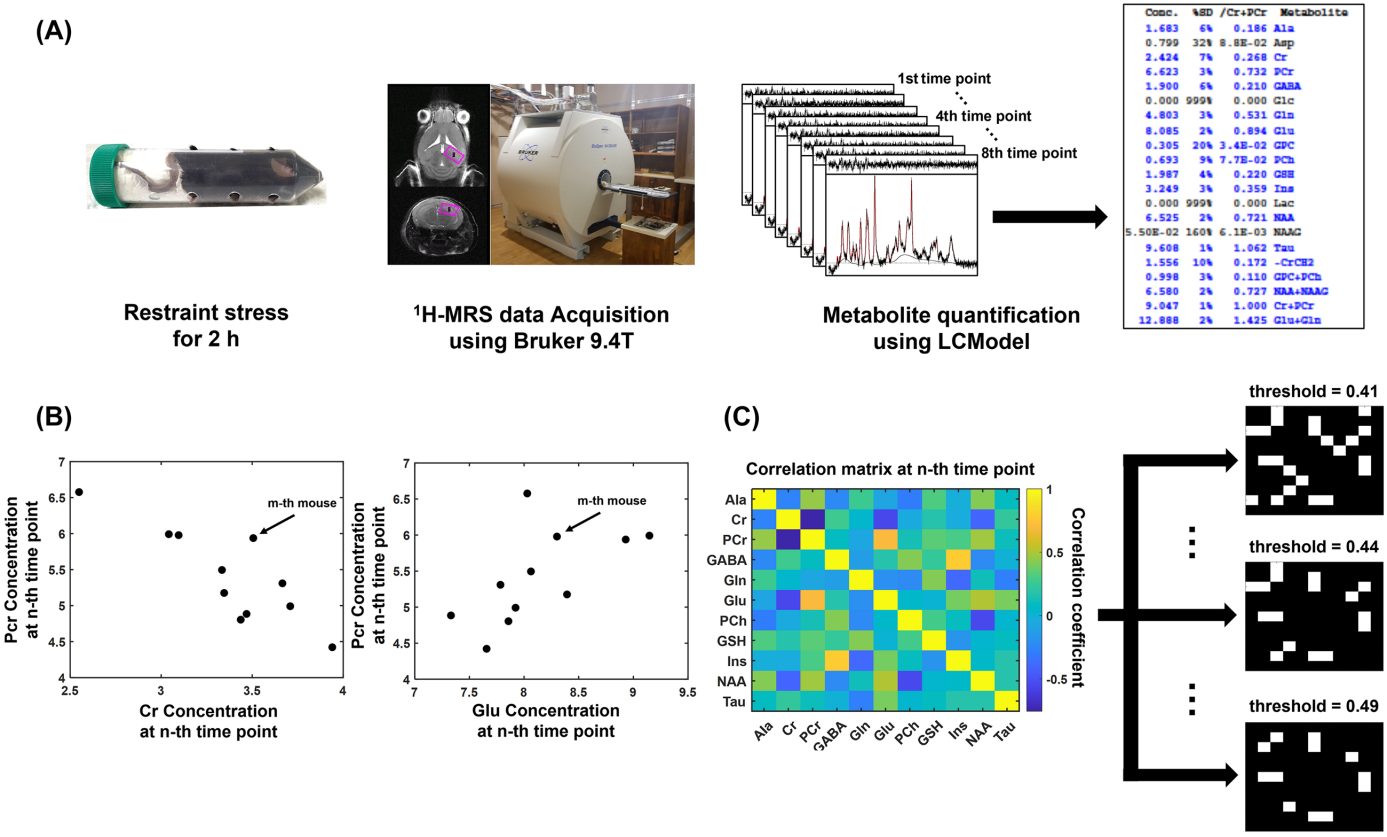
A flowchart for the construction of the metabolic network using metabolite concentration from a proton magnetic resonance spectroscopy ($^1$H-MRS). (**A**) Concentration of each metabolite from $^1$H-MRS acquired by a Bruker 9.4T scanner was estimated using LCModel. (**B**) Metabolic connection was defined as statistic association in the concentration between two metabolites. As an example of metabolic connections using concentration correlations used in the present study, we demonstrated that the concentration of PCr had a significant association with those of Cr and Glu. (**C**) The Correlation matrix was binarized by assigning one to entries above the threshold and 0 to entries under the threshold. *Ala*, alanine; *Cr*, creatine; *PCr*, phosphocreatine; *GABA*, $\gamma$-aminobutyric acid; *Glu*, glutamate; *Gln*, glutamine; *PCh*, phosphorylcholine; *GSH*, glutathione; *Ins*, myo-inositol; *NAA*, N-acetylaspartate; *Tau*, taurine.

Previous studies provided evidence that the left hippocampus is more vulnerable to stress than the right hippocampus^26,27^. McDermott et al. reported that the connectivity related to the left hippocampus was significantly correlated with both chronic stress and acute stress^26^. In addition, Rahman et al. found that the stress affected the left hippocampus volume much earlier than the right hippocampus volume^27^. Therefore, we choose the left hippocampus as voxel of interest (VOI) to investigate the dynamic change in the characteristics of the metabolic network after exposure to acute stress.

, Our study aims to provide a new approach for investigating the metabolism association between metabolites using *in vivo* $^1$H-MRS data acquired over time in the left hippocampus and to provide its quantitative information based on graph theory. In the present study, we constructed a metabolic network based on the association of metabolite concentration variations across individuals between metabolites. Then, we evaluated a SW to verify whether the constructed metabolic network is a realistic network or a random network. In addition, we applied network analysis to investigate the temporal changes in the effects of acute stress (i.e., the change in characteristics of a metabolic network including small-worldness and local efficiency of metabolite over time) after exposure to stress and to verify the biological applicability of our proposed approach. The primary hypotheses were that 1) the metabolic network for normal mice is a realistic network and 2) the metabolic network is affected by acute stress across a wide range of time. whether our metabolic network is a realistic network or a random network and 2) whether the metabolic network is affected by acute stress. To our knowledge, these approaches are the first investigations of the dynamics of metabolic connections at a network level after exposure to acute stress (Fig. 1).

## Results

### Stress effects on metabolic connections

During metabolite quantification through LCModel, two mice were excluded from analysis because they did not meet the criteria of Cramer-Rao lower bound (CRLB). Eleven mice were used in each control group and stress group for analysis.

**Figure 2:**
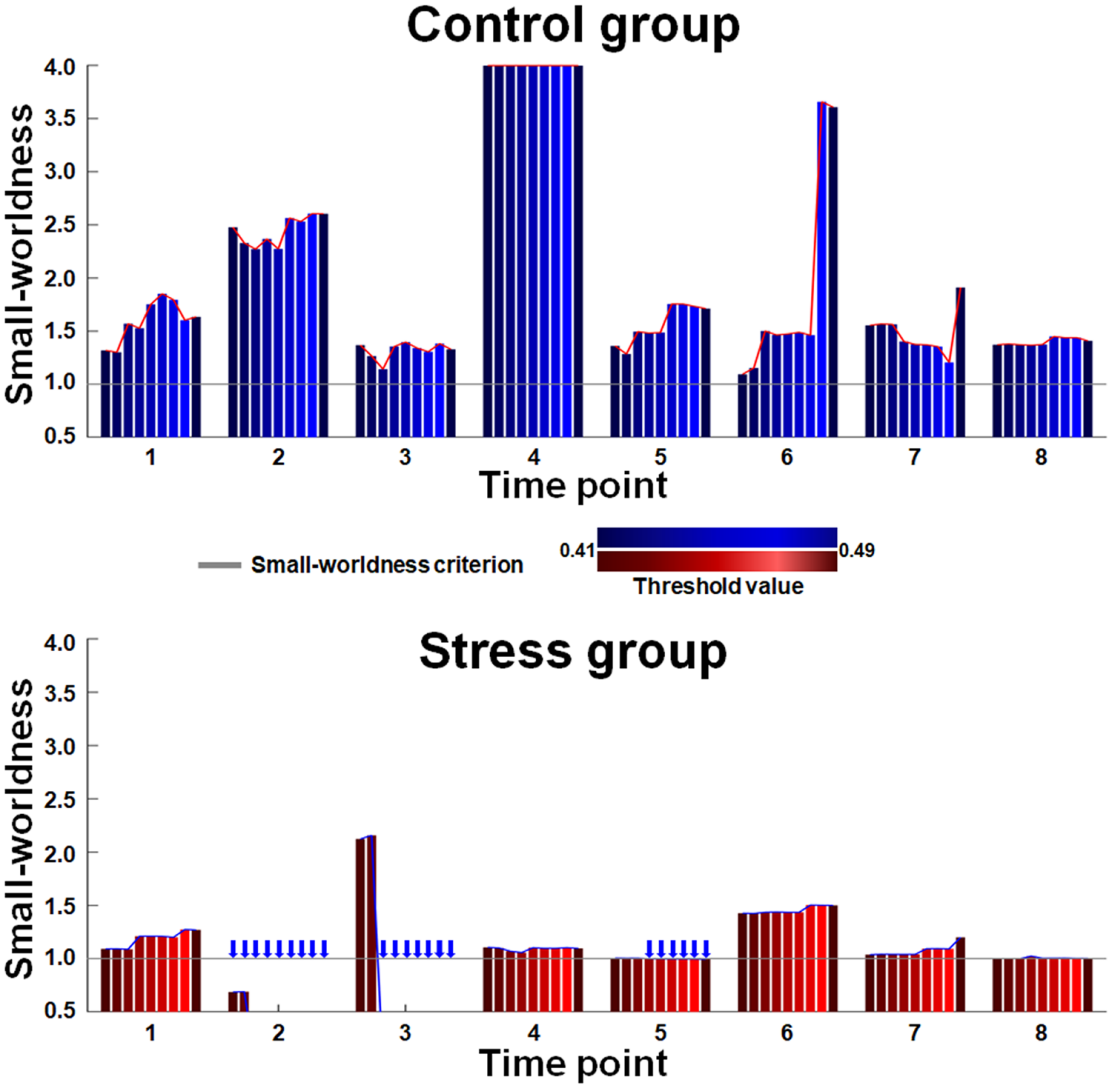
Small-worldness of metabolic networks. The color bar shows the small-worldness in control (blue) and stress (red) group. The gray line denotes the criterion of small-worldness. The blue arrow indicates that metabolic network does not meet the criterion of small-worldness at the given threshold of correlation coefficient.

**Figure 3:**
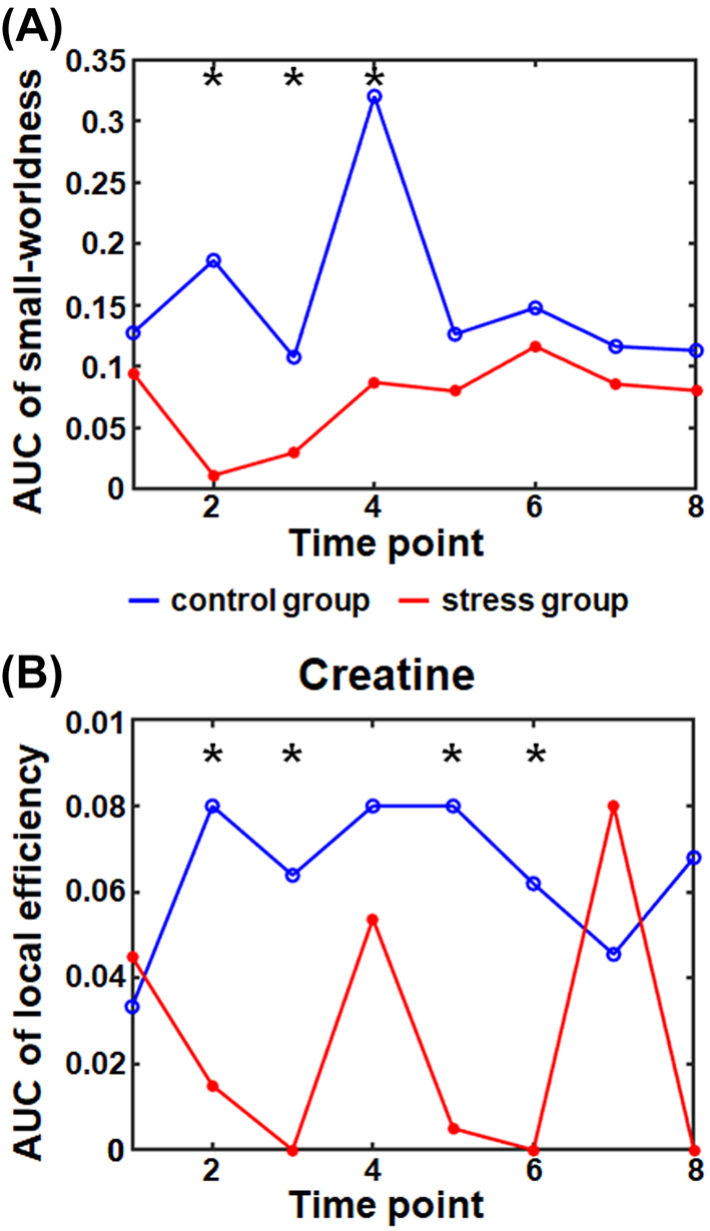
The area under the curve (AUC) of (**A**) the small-worldness and (**B**) the local efficiency of creatine in the metabolic network. The graph shows the small-worldness and local efficiency in creatine in the control (blue) and stress (red) group as a function of time. The asterisk indicates a significant difference in the AUC of the small-worldness or local efficiency of creatine (p-value < 0.05, corrected for the false discovery rate).

For the metabolic network analysis at each time point, the metabolic networks in the control group met the criterion of SW across a wide range of correlation coefficient thresholds (0.41–0.49 at a 0.01 interval) for all time points (Fig. 2). This finding suggests that the metabolic network is a real network instead of a random network and balances between the segregation and integration of metabolic function. The SW for the metabolic network in the acute stress group was also calculated across the same range of thresholds applied to the control group to investigate whether acute stress affects the metabolic network. The metabolic networks in the acute stress group did not meet the criterion of SW at second time point for thresholds of 0.41 to 0.49, third time point for thresholds of 0.43 to 0.49, and fifth time point for thresholds of 0.44 to 0.49 (Fig. 2). This suggests that the metabolic balance was disrupted after exposure to acute stress. Interestingly, after fifth time point, the metabolic network in the acute stress group tended to recover the SW and the acute stress group showed lower areas under curves (AUCs) of SW than control group at 2nd (AUC of SW = control:0.195/stress:0.010, p-value = 0.016, corrected for the false discovery rate (FDR)), 3rd (AUC of SW = control:0.105/stress:0.032, p-value = 0.037, corrected for the FDR), and 4th (AUC of SW = control:0.320/stress:0.087, p-value = 0.001, corrected for the FDR) time point. (Fig. 3A). This suggests that acute stress may cause temporary malfunction of metabolic links. For the analysis of local efficiency ($E_{local}$) at each time point, the acute stress group had lower AUCs of of creatine (Cr) within the metabolic network than the control group at the 2nd (AUC= control:0.080/stress:0.015, p-value < 0.001, corrected for the FDR), 3rd (AUC= control:0.064/stress:0.000, p-value < 0.001, corrected for the FDR), 5th (AUC= control:0.080/stress:0.005 p-value = 0.002, corrected for the FDR), and 6th (AUC= control:0.062/stress:0.000, p-value = 0.024, corrected for the FDR) time points (Fig. 3B). This means that acute stress may cause temporary Cr-related links to break.

**Figure 4:**
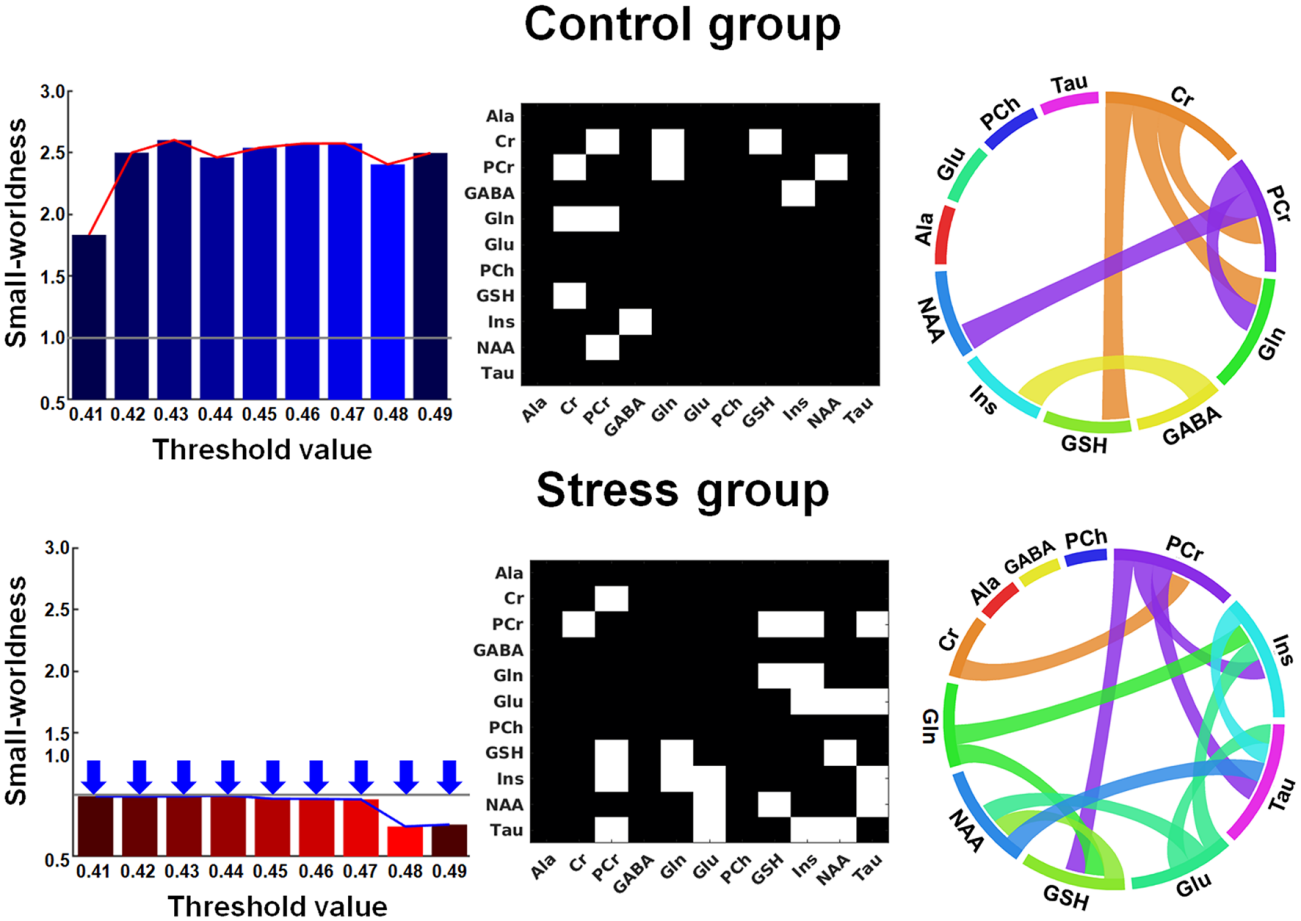
Small-worldness of the backbone metabolic networks. The color bar shows the small-worldness in control (blue) and stress (red) group. The gray line denotes the criterion of small-worldness. The blue arrow indicates that the backbone metabolic network does not meet the criterion of SW at the given threshold. The connectogram shows metabolic connections between metabolites at correlation coefficient threshold of 0.49. *Ala*, alanine; *Cr*, creatine; *PCr*, phosphocreatine; *GABA*, $\gamma$-aminobutyric acid; *Glu*, glutamate; *Gln*, glutamine; *PCh*, phosphorylcholine; *GSH*, glutathione; *Ins*, myo-inositol; *NAA*, *N*-acetylaspartate; *Tau*, taurine.

For the metabolic backbone network analysis, while the backbone network in the control group met a criterion of SW across a wide range of thresholds (0.41 to 0.49 at a 0.01 interval), the backbone network in the acute stress group did not meet the criterion. In addition, the acute stress group showed lower of SW than the control group (AUC of SW = control:0.198/stress:0.075, p-value = 0.023). This suggests that the metabolic link may not be maintained constantly over time after exposure to acute stress (Fig. 4). Furthermore, from the backbone network at highest threshold, which met the criterion of SW (i.e., threshold of correlation coefficient 0.49), this study found that Cr related-metabolic links were broken and that many metabolic links that were not clustered emerged after exposure to acute stress. For the analysis of $E_{local}$ in the metabolic backbone network, the a backbone network in acute stress group had lower $E_{local}$ of Cr (AUC = control:0.060/stess:0.000, p-value = 0.040) and higher $E_{local}$ of glutamate Glu (AUC = control:0.000/stess:0.067, p-value = 0.034) than those of a backbone network in control group. This means that acute stress may cause breaking Cr-related metabolic connections and newly forming Glu-related connections.

Constructing a backbone network over time allows investigating the presence or absence of consistent metabolic connections. In this analysis, a cluster consisted of Cr, phosphocreatine (PCr), and glutamine (Gln) in the backbone metabolic network of the control group but not in the backbone metabolic network of the stress group across all thresholds. There were Glu-related connections in the backbone metabolic network of the stress group but not in the backbone metabolic network of the control group across all threshold. In the backbone metabolic network of both the control and stress group, there was no alanine (Ala)- and phosphorylcholine (PCh)-related connections across all threshold.

## Discussion

Metabolites in the human brain exist through various metabolic processes^28^. These metabolites do not function independently but interact together with functional connections in the brain’s metabolism. To investigate the relationship between metabolites based on the $^1$H-MRS data acquired over time, the relation is a numerical value that quantifies this functional connection, and the effect of acute stress can be viewed as an objective numerical comparison. This study was motivated by a desire to overcome the limitations of cross-sectional analyses for $^1$H-MRS data. To investigate the change in the association of metabolite concentration variations between metabolites over time,$^1$H-MRS data were acquired over time, and a metabolite network was constructed by using the Spearman’s rank correlation coefficient between metabolite concentrations across individuals to verify the effects of acute stress on the left hippocampus metabolism.. The present study provides two major findings. First, the metabolic network in the stress group did not meet the criteria of small-worldness (SW) at some time points, and their SW tended to have resilience after the fifth time point, while the metabolic network in the control group met the criteria of SW at all time points. In addition, while the backbone metabolic network in the stress group did not meet the criteria of SW, the backbone metabolic network in the control group did. Many real biological, social, information and brain networks fall into the small-world network^29,30^. Since the metabolic network met the criteria of SW across a wide range of thresholds, the metabolic network can newly be considered as one of the real networks. Second, the stress group had lower local efficiency ($E_{local}$) of creatine (Cr) within the metabolic network at some time points. Similarly, a backbone network in the stress group had lower $E_{local}$ of Cr and higher $E_{local}$ of glutamate (Glu) than those of a backbone network in the control group. These findings indicate that acute stress induces the breakage of Cr-related metabolic connections and newly forming Glu-related connections and influences the homeostasis of metabolite concentration in the hippocampus.

In the present study, acute stress induced the variation in the concentration of glutamine (Gln), glutathione (GSH), and Cr. The change in concentration of these metabolites after exposure to stress can be explained by the previous findings which reported that the glucocorticoids such as corticosterone (CORT) by the hypothalamic-pituitary-adrenal (HPA) axis activity due to acute stress, induced the increase of the concentration in Gln, which was a precursor of the neurotransmitter by transporting one from the glial cell to the glutamatergic presynaptic neuron^31,32^. In addition, since GSH acts as a defense system to protect cells^33,34^ and the Cr attenuates acute stress responses through $\gamma$-aminobutyric acid (GABA)-ergic system^35^, they rapidly are secreted and consumed. This stress responses cause the breakage of the Cr-Gln and Cr-GSH connections in the stress group across a wide range of times.

Low $E_{local}$ in the metabolic network means that ability of information processing between certain metabolite and their neighbors are not efficient well. In addition, the low $E_{local}$ of Cr in the metabolic network means reduced metabolic association between Cr and other metabolites. Since the Cr has a sedative-hypnotic effect in acute stress response and may attenuate acute stress response^35^, acute stress can accelerate Cr release. Rapid release of Cr may break metabolic synchronization between Cr and other metabolites. It might cause reduced $E_{local}$ of Cr in the metabolic network of the stress group across a wide range of time points and also backbone metabolic network. In the backbone network of the stress group, there were Glu-related metabolic connections, but not in the backbone network of the control group. Since HPA axis activity after exposure to acute stress accelerates the Glu release, it might cause new metabolic synchronization between Glu and other metabolites which led to increased $E_{local}$ of Glu in contrast with Cr.

In a network, a specialized function and segregated processing are based on the concept of cluster or connected triangle^22^. In the backbone metabolic network analysis, a cluster consisted of Gln, Cr, and phosphocreatine (PCr) in the control group, but there was no cluster in the stress group across a wide range of thresholds. Therefore, we focused on interpreting this cluster to understand the change in metabolic association due to acute stress responses. The Cr and PCr are directly related through the creatine kinase (CK) (i.e., PCr shuttle system). The PCr is decomposed into Cr and inorganic phosphate for adenosine triphosphate (ATP) formation through the CK. Since ATP hydrolysis results in products of adenosine diphosphate (ADP) and inorganic phosphate, it is re-synthesized into PCr through the CK^36^. The Cr and Gln are indirectly related through the Glu-Gln cycling. The Cr enhances the energy-consuming (i.e., convert from ATP to ADP) conversion of Glu to Gln through Glu-Gln cycling^37^. According to this phenomenon, there is a cluster that consists of metabolic connections between Cr and PCr, Gln and Cr, and Gln and Cr in the control group. Since the Cr has a sedative-hypnotic effect in acute stress response^35^, acute stress can accelerate Cr release. In addition, the Glu from the presynaptic neuron is released upon the HPA axis activity after exposure to stress^38^. Because the released Glu has neurotoxicity, it travels to nearby astrocytes and is converted to the Gln by consuming energy^39^. The converted Gln is back transported to the presynaptic neuron. Consequently, the acute stress caused alteration in the concentration of Cr, PCr and Gln that may have led to a break in the Gln-Cr-PCr cluster after exposure to acute stress. Furthermore, although the metabolic network in the stress group was not a SW network, remarkable Glu-related connections emerged in the present study. The acute stress activates the HPAaxis^10^. The hypothalamus releases the corticotropin-releasing hormone, which stimulates the pituitary secretion of the adrenocorticotropic hormone. It activates CORT release from the adrenal glands^38,40,41^. CORT enters the hippocampus via the bloodstream and combines with the glucocorticoid receptors to release an excitatory neurotransmitter, Glu, from the presynaptic membrane of the neuron^39^. In particular, Glu-Gln cycling in the hippocampus of the mouse brain can be described as the transport of Glu acting as the excitatory neurotransmitter and Gln forming through the amidation of Glu in the tripartite synapses^42,43^. Thus, Gln production and Glu decomposition occur in astrocytes, and the opposite effects occur in neurons via Glu-Gln cycling^44^. These reasons explain that the connection between Gln-Cr and Gln-PCr broke after exposure to acute stress. There is evidence for forming Glu-related connections in the stress group. Many kinds of stress affect the metabolites in the glutamatergic and aspartate systems through the tricarboxylic acid (TCA)-cycle^45^. The Glu interacts with $\alpha$-ketoglutarate in the TCA-cycle and it can be caused by the effect of the protein which makes up the TCA-cycle. Furthermore, the energy required for Glu cycling in Glu-Gln cycling between astrocytes and neurons can be estimated as three adenosine triphosphate per Glu molecule^39,46,47^. It can be caused by N-acetylaspartate (NAA) produced from the aspartate and acetate, and it is synthesized with adenosine triphosphate and converted to acetyl-coenzyme A. Finally, this synthesis affects the aminotransferase, which reacts with [aspartate + $\alpha$-ketoglutarate] to form [Glu + oxaloacetate]^48^. This reaction, which results in increasing Glu concentration, may induce an increase in NAA concentration, and in consequence, Glu is statistically connected with NAA.

In the present study, our findings propose that acute stress induces an imbalance between metabolite variations, which forms random metabolic connections in the left hippocampus, and it needs a period to return to the pre-stress level. Although it is a natural stress response, previous studies have reported changes in the metabolism and brain structure of the hippocampus due to stress. The acute stress induces increases in extracellular Glu, and it may thus underlie the dendritic remodeling in the hippocampus^6,49^. Interestingly, one study proposed that stress causes the change in metabolism, including glucocorticoids and serotonin acting through excitatory amino acids, which may be a mediator of hippocampus atrophy^50^, and that glucocorticoids can influence neurotransmission such as Glu and $\gamma$-aminobutyric acid through crosstalk with the endocannabinoid system^51^. Analysis of the metabolic network can provide evidence of metabolic imbalance, which may be a pre-stage of alterations to the brain structure due to stress, and additional research is required to investigate the stress response in the hippocampus.

The main strengths of this study were 1) the mouse age, gender and strain homogeneity, which reduced associated confounding with the metabolites and stress response that could change depending on the above conditions and 2) $^1$H-MRS data acquired over consecutive times to analyze the change for concentrations of metabolites in the left hippocampus. However, the present study had several limitations. First, the number of mice was insufficient to satisfy normality and determine real connections between metabolites. To minimize this, we applied statistical analysis using the sign test to determine whether there is the connection for each pair of metabolites across all mice. Second, the time resolution (i.e., about 17 minutes interval between time points) was insufficient to measure the real-time dynamics of the occurring metabolism in the left hippocampus. Finally, in the present study, we did not investigate the effects that may be induced by a combination of restraint and anesthesia. Anesthesia might affect our measurement. Nevertheless, anesthesia of experimental animals is essential to obtain $^1$H-MRS data with minimal movement and stress and high reproducibility^52^. Isoflurane-based inhalation anesthesia was applied to minimize the accumulation of anesthetic metabolites and the harmful effects for long-term use of the anesthetic, and it makes inhalation anesthesia with isoflurane possible for 8 hours since no practical difference occurs^53^. Since all experimental conditions were made the same, the comparison between groups can be considered as an analysis for the effect of restraint stress. Although this is beyond the scope of the current study, it remains an important line of inquiry for future research. Despite these limitations, the present study provided a non-invasive measurement of dynamic change in the connection between metabolites after exposure to acute stress.

In summary, we focused on the correlation coefficient between the metabolite concentrations (i.e., synchronization of metabolites) after exposure to acute stress. Although it remains unclear how an individual-level metabolic network might be constructed directly from $^1$H-MRS data, the group-level metabolic networks have provided a statistical framework to study synchronized metabolic changes in the left hippocampus across the population. Constructing a group-level metabolic network may reflect desynchronization or synchronization of metabolites due to stress responses. In this study, we found that the metabolic network is a realistic network rather than a random network and that the Cr-related connection is temporarily broken while the Glu-related connection is newly created. The present study demonstrated that the metabolic network analysis based on $^1$H-MRS obtained over time could be an effective tool to non-invasively examine broken metabolic balance in the left hippocampus after exposure to acute stress. Although preliminary, our findings suggest that our approach may be used to understand what causes brain abnormality in stress-induced humans.

## Methods

### Experimental animals

Male C57BL/6N mice (ORIENT BIO Inc., South Korea) with body weight of 18-25 g at the age of four weeks were used as experimental animals. The mice were housed three per transparent plastic cage and were allowed to acclimate for two weeks before $^1$H-MRS experiment to adjust to the experimental environment and minimize stress from the unfamiliar situation. The mice had unconstrained access to water and food, and consistent conditions were maintained in temperature (21–23 $^\circ$C) and humidity (50–60%), with 12h/12h light/dark cycle (light on from 06:00 to 18:00). Since data was acquired for three mice were per day, restraint stress was applied for 2 hours in the light phase at 08:00, 11:00, and 14:00. The effect of restraint stress applied at different times is related to the circadian rhythm of stress hormone (i.e., corticosterone) during 24 hours in control animals^54^. In the light phase (08:00-14:00), the changes in corticosterone of control animal are insignificant^55^ as well as the level of corticosterone that is increased by restraint stress is much higher than that by changes in the circadian rhythm^56^. Control animals were kept in the cages without removal of food during the period of restraint stress because food deprivation can be a stressor. The mice were randomly divided into a control group (n = 12) and a stress group (n = 12).

All experiments were approved by the Institutional Animal Care and Use Committee and conducted at Lee Gil Ya Cancer and Diabetes Institute accredited in Association for Assessment and Accreditation for Laboratory Animal Care International (AAALAC), which is the international standard for animal care and use globally. All procedures for experiments complied with the Center of Animal Care and Use (CACU) for Animal Research (Guidelines for Animal Users). In addition, the study was carried out following the ARRIVE guidelines (https://arriveguidelines.org)^57^.

### Restraint stress protocol

Based on the stress protocol of previous studies^58,59^, 12 mice were physically exposed to acute restraint stress for 2 h in a 50 mL conical tube (3 cm in diameter and 10 cm in length). The holes of the plastic tube toward the body (3 mm in diameter) and mouth (5 mm in diameter) of a mouse were made to promote airflow for ventilation and kept intact with the background noise in the air-conditioned room for the restraint procedure (Fig. 1A). To minimize mouse movement for the purpose of perfect immobility in the tube, the residual space in the tube with the mouse was filled with something that could pressurize the body and head of the mouse to fix them closely. The mouse was used for *in vivo* $^1$H-MRS data acquisition as soon as the procedure of restraint was completed. The mice in the control group were never exposed to restraint stress and remained in the cage until the beginning of data acquisition.

### Proton magnetic resonance spectroscopy

A 9.4T Bruker BioSpec Avance III 94/20 USR (Bruker BioSpin MRI GmbH, Ettlingen, Germany) was used for data acquisition. To localize a voxel of interest (VOI) in a mouse brain, T2-weighted MR images were acquired using rapid acquisition with relaxation enhancement and the following acquisition parameters: TR = 5000 ms, TE$_{eff}$ = 48 ms, field of view (FOV) = 3 $\times$ 3 cm$^2$, matrix size = 256 $\times$ 256, slice thickness = 1 mm, VOI size = 1.8 $\times$ 3.4 $\times$ 1.8 mm$^3$, 11.016 $\mu$L. The VOI was placed in the left hippocampus, which is associated with acute stress (Fig. 1A). Fast, automatic shimming technique by mapping along projections (FASTMAP) was performed to improve the homogeneity of the magnetic field in the VOI and was repeated until the linewidth of the resulting water in the same VOI was below 14 Hz. Based on single-voxel spectroscopy, $^1$H-MRS data were acquired using a point-resolved spectroscopy with the following acquisition parameters: TR = 4000 ms, TE = 10 ms, number of averages = 512, complex points = 4096, spectral width = 5000 Hz. Variable power RF pulses suppressed the water signal with optimized relation delays (VAPOR). Suppression of the unwanted signal outside the VOI was performed by outer volume suppression. The unsuppressed water signal was also obtained at the same VOI to correct eddy-current-induced distortion during acquisition and to obtain absolute quantification of the metabolites. The relaxation effect is not considered due to long TR and short TE^60^.

To investigate dynamic metabolic changes over time, *in vivo* $^1$H-MRS experiment in both groups was performed alternatively for 8 days on three mice per day. All data were collected every 34 minutes at four consecutive time points in both groups excluding anesthesia, voxel positioning, shimming, and the acquisition of the unsuppressed water signal. Mice that were spontaneously respiring were anesthetized with isoflurane (4.0% at introduction and 1.0–2.0% during the experiment) in a 1:2 O$_{2}$:air mixture that was delivered to the mouth using an anesthesia apparatus. Mice were placed on a flat mouse bed in a prone position and the brain was tightly fixed with a bite bar and two ear inserts. To maintain a stable body temperature at 38 $^\circ$C during anesthesia, a water-heated body-warming system was used. To adjust the anesthetic concentration for the stability of the mouse’s condition, the rate of respiration was consecutively monitored using MR-compatible instruments (SA instruments, Inc., Stony Brook, NY, USA).

### Metabolite quantification

The method for determining the intensity of the spectral peaks by LCModel used for metabolite quantification in the present study is as follows: First, it is a model that includes not only the baseline (including macromolecules) but also the spectra obtained from the phantom solution of each metabolite to be included as a basis set known as prior knowledge such as concentration and chemical shift. This model should contain spectra of very high quality and should have the same protocol as the *in vivo* spectra to be analyzed later. In addition, since it is specific to MRI (e.g., protocol, field strength) and region (e.g., VOI), the model must be recreated if the conditions of the *in vivo* spectrum to be analyzed change. The second is to acquire unsuppressed water signals for absolute quantification of metabolites from *in vivo* spectra. Third, it is possible to estimate the intensity of each metabolite by fitting it to the *in vivo* spectrum using the constrained nonlinear least-squares algorithm based on the basis set and the zero- and first-order phase correction^61,62^.

To correct the frequency and phase shift caused by a long scan time and acquire a series of $^1$H-MRS data consisting of 512 free induction decay (FID) signals, a reference data through which frequency- and phase-shifted values were calculated was determined by finding the FID signal with the smallest difference from each FID signal using mean squared error after extracting the median value from each data point for the 512 FID signals using FID-A software based on MATLAB^63,64^. These corrections were repeated once for each $^1$H-MRS data and once for the data set. The corrected 512 FID signals were divided into two of 256 FID signals to improve the temporal resolution for metabolic network construction (i.e., about 17 minutes interval between time points).

The concentrations of metabolites were calculated by fitting the experimentally obtained $^1$H-MRS based on a simulated basis-set using the LCModel software (Fig. 1A)^61,65^. Of the 17 metabolites included in the simulated basis-set, the 11 metabolites that met the Cramer-Rao lower bound (CRLB) of < 35% at all time points were included in the analysis: alanine (Ala), creatine (Cr), phosphocreatine (PCr), $\gamma$-aminobutyric acid (GABA), glutamate (Glu), glutamine (Gln), phosphorylcholine (PCh), glutathione (GSH), myo-inositol(Ins), N-acetylaspartate (NAA), and taurine (Tau). Although N-acetyl-aspartylglutamate, glycine, and serine are theoretically possible in common, it is difficult to quantify in the brain experimentally because their concentrations are minimal^66^.

### Metabolic network construction

The metabolic network construction was motivated by the cortical network construction and used the correlation coefficient of cortical thickness between every pair of brain regions, which has been used to characterize the organization of cortical brain networks^30,67^. Usually, Pearson’s correlation analysis is conducted when the assumptions of this test including normality distribution and both variables measurement are met^68^. However, our data did not meet the normality distribution and small sample size. Thus, to characterize the relationships between metabolites, we employed Spearman’s rank correlation analysis which is another common statistical correlation method that could be adopted if Pearson’s correlation analysis assumptions are not met^68^. This study first defined a metabolic connection as statistical associations measured by computing Spearman’s rank correlation coefficient of mice between the concentrations of every pair of metabolites (Fig. 1B). In addition, we constructed the backbone metabolic network which consisted of the most consistent connections over time. Since we focused on an on-off (i.e., 0 or 1) connection pattern, we did not consider the negative sign and used the absolute value of Spearman’s rank correlation coefficient. Thus, a non-parametric one-tailed sign test was applied to identify the highly consistent metabolic connections. For each pair of metabolites, the sign test was performed with the null hypothesis that there is no existing connection (i.e., absolute Spearman’s rank correlation coefficient = 0) and the alternative hypothesis that there is an existing connection (i.e., absolute Spearman’s rank correlation coefficient > 0). To reject the null hypothesis, we set the significance level alpha to 0.025 for a non-parametric one-tailed sign test. Finally, this study obtained an 11 by 11 inter-metabolic correlation matrix (i.e., metabolic network, Fig. 1C) at each time point and 11 by 11 consistent metabolic connection matrix (i.e., backbone metabolic network).

The inter-metabolic correlation matrix and the consistent metabolic connection matrix were binarized, assigning 1 to entries above the threshold and 0 to entries under the threshold. There is currently no gold standard to select a single threshold for constructing a network^67^. To reduce the connection by chance and the analysis bias due to selecting a single correlation coefficient threshold, in the present study, we applied a wide range of correlation coefficient thresholds (0.41 to 0.49 at a 0.01 interval) (Fig. 1C). The minimum and maximum thresholds were chosen because the networks of the control group met the small-worldness criterion across all time points (Fig. 2). Both control and stress groups did not meet the small-worldness criterion at the other thresholds.

### Small-world network

In the present study, to investigate small-world characteristics, we used efficiency metrics, since the path length is defined as infinite between disconnected nodes. In the previous study, to avoid the computational nuisance in dealing with infinite path lengths, the path length of a disconnected node was set to the maximum path length between any pair of nodes^69^. Thus, it may not be a meaningful global measure for the sparse network. An alternative measurement can be global efficiency ($E_{global}$) to measure network integration and an infinite path length corresponds to $E_{global}$ of zero^70^. The present study calculated 1) the global efficiency ($E^{real}_{global}$) as a measure of functional integration and local efficiency ($E^{real}_{local}$) as a measure of functional segregation, and 2) the mean global efficiency ($E^{rand}_{global}$) and local efficiency ($E^{rand}_{local}$) of the 1000 matched random networks with the same number of nodes, mean degree, and degree distribution as the real network. $E_{global}$ is defined as the inverse of mean path length, which is the minimum number of edges between any pair of nodes, and $E_{local}$ is defined as the efficiency of the connections between the nearest neighborhoods of the node^25,71^.

A real network is considered a small-world network that satisfies the following conditions: 1) normalized global efficiency [$\gamma$ $=$ $E^{real}_{global} / E^{rand}_{global}$ $\approx 1$] and 2) normalized local efficiency [$\lambda$ $=$ $mean(E^{real}_{local}) / mean(E^{rand}_{local})$ $> 1$] (i.e., small-worldness (SW) [$\sigma$ $=$ $\lambda$ / $\gamma$ $> 1$]). SW indicates the optimized balance between functional segregation and integration in the network^25^. To examine stress effects on the variance of individual metabolite and on metabolic connections over time, the present study compared the $E_{local}$ and SW between groups, respectively.

### Statistical analysis

A non-parametric permutation test was applied to determine the statistical significance of the differences in the SW between groups^67,72^. To test the null hypothesis that the observed group differences could occur by chance, this study randomly reallocated a set of metabolite concentrations in each mouse to the control group or stress group, recomputed the correlation matrix for each randomized group, and then obtained the corresponding binarized matrix using the same correlation threshold. Next, this study calculated the SW for each randomized group and obtained the differences between the randomized groups. This randomization procedure was repeated 1000 times at each correlation threshold value. The 95 percentile points of each distribution were used as the critical values for a non-parametric permutation test of the null hypothesis with a probability of type I error of 0.05. The p-values were adjusted for multiple comparisons using false discovery rate (FDR) correction. Group differences in the areas under curves (AUCs) were assessed at all correlation thresholds.

## References

[1] Selye, H. *The Stress of Life* (McGraw-Hill, 1956).

[2] Musazzi L, Tornese P, Sala N, Popoli M (2017). Acute or chronic? A stressful question. Trends Neurosci..

[3] Dhabhar F (2018). The short-term stress response-mother nature’s mechanism for enhancing protection and performance under conditions of threat, challenge, and opportunity. Front. Neuroendocrinol..

[4] Feder A, Nestler E, Charney D (2009). Psychobiology and molecular genetics of resilience. Nat. Rev. Neurosci..

[5] Lee M (2016). Temporal variability of glucocorticoid receptor activity is functionally important for the therapeutic action of fluoxetine in the hippocampus. Mol. Psychiatry.

[6] McEwen B (1999). Stress and hippocampal plasticity. Annu. Rev. Neurosci..

[7] Musazzi L, Tornese P, Sala N, Popoli M (2018). What acute stress protocols can tell us about ptsd and stress-related neuropsychiatric disorders. Front. Pharmacol..

[8] McEwen B, Gianaros P (2011). Stress- and allostasis-induced brain plasticity. Annu. Rev. Med..

[9] Conrad, C., Wright, R. & McLaughlin, K. *Stress and vulnerability to brain damage* Encyclopedia of Neuroscience (Academic Press, 2009).

[10] McEwen B (2015). Mechanisms of stress in the brain. Nat. Neurosci..

[11] De Kloet E, Joëls M, Holsboer F (2005). Stress and the brain: From adaptation to disease. Nat. Rev. Neurosci..

[12] De Kloet E, DeRijk R, Meijer O (2007). Therapy insight: Is there an imbalanced response of mineralocorticoid and glucocorticoid receptors in depression?. Nat. Clin. Pract. Endocrinol. Metab..

[13] Popoli M, Yan Z, McEwen B, Sanacora G (2012). The stressed synapse: The impact of stress and glucocorticoids on glutamate transmission. Nat. Rev. Neurosci..

[14] Kim, S.-Y. *et al.* Acute restraint-mediated increases in glutamate levels in the rat brain: An in vivo $$^1$$h-mrs study at 4.7t. *Neurochem. Res.* **37**, 740–748 (2012). 10.1007/s11064-011-0668-y 22187117

[15] Yoo C, Lim S, Song K, Woo D, Choe B (2018). Investigating the metabolic alterations in a depressive-like rat model of chronic forced swim stress: An in vivo proton magnetic resonance spectroscopy study at 7t. Neurochem. Int..

[16] Jahagirdar, S., Suarez-Diez, M. & E., S. Simulation and reconstruction of metabolite-metabolite association networks using a metabolic dynamic model and correlation based algorithms. *J. Proteome Res.* **18**, 1099–1113 (2019). 10.1021/acs.jproteome.8b00781 30663881

[17] Albert R, Barabási A (2002). Statistical mechanics of complex networks. Rev. Mod. Phys..

[18] Scott J (1988). Social network analysis. Sociology.

[19] Zhang W, Wang S, Tian X, Yu D, Yang Z (2017). The backbone of urban street networks: Degree distribution and connectivity characteristics. Adv. Mech. Eng..

[20] Haggarty S, Clemons P, Schreiber S (2003). Chemical genomic profiling of biological networks using graph theory and combinations of small molecule perturbations. J. Am. Chem. Soc..

[21] Sporns O, Chialvo D, Kaiser M, Hilgetag C (2004). Organization, development and function of complex brain networks. Trends Cogn. Sci..

[22] Rubinov M, Sporns O (2010). Complex network measures of brain connectivity: Uses and interpretations. Neuroimage.

[23] Watts D, Strogatz S (1998). Collective dynamics of ‘small-world’ networks. Nature.

[24] Barrat A, Weigt M (2000). On the properties of small-world network models. Eur. Phys. J. B..

[25] Latora V, Marchiori M (2001). Efficient behavior of small-world networks. Phys. Rev. Lett..

[26] McDermott K, Ren P, Lin F (2019). The mediating role of hippocampal networks on stress regulation in amnestic mild cognitive impairment. Neurobiol. Stress.

[27] Rahman MM, Callaghan CK, Kerskens CM, Chattarji S, O’Mara SM (2016). Early hippocampal volume loss as a marker of eventual memory deficits caused by repeated stress. Sci. Rep..

[28] Falkowska A (2015). Energy metabolism of the brain, including the cooperation between astrocytes and neurons, especially in the context of glycogen metabolism. Int. J. Mol. Sci..

[29] Humphries, M. D. & Gurney, K. Network ‘small-world-ness’: A quantitative method for determining canonical network equivalence. *PLoS ONE* **3**, 266 (2008). 10.1371/journal.pone.0002051 PMC2323569 18446219

[30] He Y, Chen Z, Evans A (2007). Small-world anatomical networks in the human brain revealed by cortical thickness from mri. Cereb. Cortex.

[31] Albrecht J, Sidoryk-Wegrzynowicz M, Zielinska M, Aschner M (2010). Roles of glutamine in neurotransmission. Neuron Glia Biol..

[32] Popoli, M., Yan, Z., McEwen, B. & Sanacora, G. The stressed synapse: The impact of stress and glucocorticoids on glutamate transmission. *Nat. Rev. Neurosci.* **13**, 22–37 (2011). 10.1038/nrn3138 PMC3645314 22127301

[33] Maher P (2005). The effects of stress and aging on glutathione metabolism. Ageing Res. Rev..

[34] Ghizoni D (2006). Alterations in glutathione levels of brain structures caused by acute restraint stress and by nitric oxide synthase inhibition but not by intraspecific agonistic interaction. Behav. Brain Res..

[35] Koga Y, Takahashi H, Oikawa D, Denbow DM, Furuse M (2005). Brain creatine functions to attenuate acute stress responses through gabanergic system in chicks. Neuroscience.

[36] Fedosov, S. N. Creatine–creatine phosphate shuttle modeled as two-compartment system at different levels of creatine kinase activity. *Biochim. Biophys. Acta.* **1208**, 238–246 (1994). 10.1016/0167-4838(94)90109-0 7947954

[37] Bender, A. *et al.* Creatine supplementation lowers brain glutamate levels in Huntington’s disease. *J. Neurol.* **252**, 36–41 (2005). 10.1007/s00415-005-0595-4 15672208

[38] Kudielka B, Schommer N, Hellhammer D, Kirschbaum C (2004). Acute hpa axis responses, heart rate, and mood changes to psychosocial stress (tsst) in humans at different times of day. Psychoneuroendocrinology.

[39] Osborne D, Pearson-Leary J, McNay E (2015). The neuroenergetics of stress hormones in the hippocampus and implications for memory. Front. Neurosci..

[40] Kudielka, B. & Kirschbaum, C. Sex differences in hpa axis responses to stress: A review. *Biol. Psychol.* **69**, 113–132 (2005). 10.1016/j.biopsycho.2004.11.009 15740829

[41] Russell, A. *et al.* Factors promoting vulnerability to dysregulated stress reactivity and stress-related disease. *J. Neuroendocrinol.* **30**, (2018). 10.1111/jne.12641 PMC6181794 30144202

[42] Bröer S, Brookes N (2001). Transfer of glutamine between astrocytes and neurons. J. Neurochem..

[43] Albrecht, J., Sonnewald, U., Waagepetersen, H. & Schousboe, A. Glutamine in the central nervous system: Function and dysfunction. *Front. Biosci.* **12**, (2007). 10.2741/2067 17127302

[44] Somersalo, E. & Calvetti, D. Quantitative in silico analysis of neurotransmitter pathways under steady state conditions. *Front. Endocrinol.* **4**, 137 (2013). 10.3389/fendo.2013.00137 PMC3792486 24115944

[45] Xu, S., Liu, Y., Pu, J. & Gui, S. Chronic stress in a rat model of depression disturbs the glutamine-glutamate-gaba cycle in the striatum, hippocampus, and cerebellum. *Neuropsychiatr. Dis. Treat.* **16**, 557–570 (2020). 10.2147/NDT.S245282 PMC7047974 32158215

[46] Attwell D, Laughlin S (2001). An energy budget for signaling in the grey matter of the brain. J. Cereb. Blood Flow Metab..

[47] Harris J, Jolivet R, Attwell D (2012). Synaptic energy use and supply. Neuron.

[48] Clark, J. *N*-acetylaspartate as a reservoir for glutamate. *Med. Hypoth.* **67**, 506–512 (2006). 10.1016/j.mehy.2006.02.047 16730130

[49] Lowy M, Gault L, Yamamoto B (1993). Adrenalectomy attenuates stress-induced elevations in extracellular glutamate concentrations in the hippocampus. J. Neurochem..

[50] Bremner, J. Does stress damage the brain?. *Biol. Psychiatry* **45**, 797–805 (1999). 10.1016/s0006-3223(99)00009-8 10202566

[51] Katona I, Freund T (2008). Endocannabinoid signaling as a synaptic circuit breaker in neurological disease. Nat. Med..

[52] Lukasik, V. M. & Gillies, R. J. Animal anaesthesia for in vivo magnetic resonance. *NMR Biomed.* **16**, 459–467 (2003). 10.1002/nbm.836 14696002

[53] Tremoleda, J. L., Kerton, A. & Gsell, W. Anaesthesia and physiological monitoring during in vivo imaging of laboratory rodents: Considerations on experimental outcomes and animal welfare. *EJNMMI Res.* **2**, 44 (2012). 10.1186/2191-219X-2-44 PMC3467189 22877315

[54] Koch CE, Leinweber B, Drengberg BC, Blaum C, Oster H (2017). Interaction between circadian rhythms and stress. Neurobiol. Stress.

[55] Dallman, M. F., Akana, S. F., Bell, M. E. & Strack, A. M. Bottomed out: Metabolic significance of the circadian trough in glucocorticoid concentrations. *Int. J. Obes. Relat. Metab. Disord.* **24**, S40–S46 (2000). 10.1038/sj.ijo.0801276 10997607

[56] McClennen SJ, Cortright DN, Seasholtz AF (1998). Regulation of pituitary corticotropin-releasing hormone-binding protein messenger ribonucleic acid levels by restraint stress and adrenalectomy. Endocrinology.

[57] du Sert, N. P. *et al.* Reporting animal research: Explanation and elaboration for the arrive guidelines 2.0. *PLoS Biol.* **18**, e3000411 (2020). 10.1371/journal.pbio.3000411 PMC7360025 32663221

[58] Bonneau R, Sheridan J, Feng N, Glaser R (1993). Stress-induced modulation of the primary cellular immune response to herpes simplex virus infection is mediated by both adrenal-dependent and independent mechanisms. J. Neuroimmunol..

[59] Iwakabe K (1998). The restraint stress drives a shift in th1/th2 balance toward th2-dominant immunity in mice. Immunol. Lett..

[60] Provencher, S. W. LCModel & LCMgui User’s Manual (2020). Accessed 27 May 2020.

[61] Provencher S (1993). Estimation of metabolite concentrations from localized in vivo proton nmr spectra. Magn. Reson. Med..

[62] Provencher SW (1982). A constrained regularization method for inverting data represented by linear algebraic or integral equations. Comput. Phys. Commun..

[63] Near J (2015). Frequency and phase drift correction of magnetic resonance spectroscopy data by spectral registration in the time domain. Magn. Reson. Med..

[64] Simpson R, Devenyi G, Jezzard P, Hennessy T, Near J (2017). Advanced processing and simulation of mrs data using the fid appliance (fid-a)-an open source, matlab-based toolkit. Magn. Reson. Med..

[65] Provencher, S. Automatic quantitation of localized in vivo 1h spectra with lcmodel. *NMR Biomed.* **66**, 260–264 (2001). 10.1002/nbm.698 11410943

[66] Govindaraju V, Young K, Maudsley AA (2000). Proton nmr chemical shifts and coupling constants for brain metabolites. NMR Biomed..

[67] He, Y., Chen, Z. & Evans, A. Structural insights into aberrant topological patterns of large-scale cortical networks in Alzheimer’s disease. *J. Neurosci.* **28**, 4756–4766 (2008). 10.1523/JNEUROSCI.0141-08.2008 PMC6670444 18448652

[68] Ong MHA, Puteh F (2017). Quantitative data analysis: Choosing between spss, pls, and amos in social science research. Int. Interdiscip. J. Sci. Res..

[69] Zalesky A (2010). Whole-brain anatomical networks: Does the choice of nodes matter?. Neuroimage.

[70] Wang J (2009). Parcellation-dependent small-world brain functional networks: A resting-state fmri study. Hum. Brain Mapp..

[71] Achard S, Bullmore E (2007). Efficiency and cost of economical brain functional networks. PLoS Comput. Biol..

[72] Bullmore E (1999). Global, voxel, and cluster tests, by theory and permutation, for a difference between two groups of structural mr images of the brain. IEEE Trans. Med. Imaging.

